# Canine Blood Group Prevalence and Geographical Distribution around the World: An Updated Systematic Review

**DOI:** 10.3390/ani11020342

**Published:** 2021-01-29

**Authors:** Sara Mangiaterra, Giacomo Rossi, Maria Teresa Antognoni, Matteo Cerquetella, Andrea Marchegiani, Arianna Miglio, Alessandra Gavazza

**Affiliations:** 1 School of Biosciences and Veterinary Medicine, University of Camerino, 62032 Camerino, Italy; sara.mangiaterra@unicam.it (S.M.); giacomo.rossi@unicam.it (G.R.); matteo.cerquetella@unicam.it (M.C.); andrea.marchegiani@unicam.it (A.M.); 2 Department of Veterinary Medicine, Blood Bank and Transfusion Unit EMOVET-UNIPG, University of Perugia, 06126 Perugia, Italy; maria.antognoni@unipg.it (M.T.A.); miglioarianna@libero.it (A.M.)

**Keywords:** systematic review, canine blood groups, countries, blood-typing assay, prevalence

## Abstract

**Simple Summary:**

“Blood group” or “blood type” refers to the blood group system comprising red blood cell antigens and a specific pattern. Many studies have characterized dog blood groups by the prevalence of the Dog Erythrocyte Antigen (DEA), Kai, and Dal antigens in different geographic areas and by using a variety of methods. Some pioneering studies on blood groups, upon which others were subsequently based, were conducted by Bowdler, Colling, and Hall in the 1970s and 1980s. Our results show that most relevant studies covered the European and American continents, and the methods used to identify DEA, Kai, and Dal groups evolved from 1999 to 2020, albeit without a single method based upon specificity and sensitivity. However, the existence of rapid commercial kits for the determination of the DEA 1 group makes this group the most widely used in clinical practice. Through a systematic review, our aim was to illustrate the countries in the world where different blood groups have been identified with reference to the different methods used and the prevalence of those groups among dog breeds.

**Abstract:**

In recent years, blood transfusions have been more commonly given to pets. The importance of determining blood groups in dogs and cats is, therefore, well-known for reducing the risk of adverse reactions in the recipient blood caused by a “non-compatible” donor. This systematic review summarizes data from previously published reports and follows the PRISMA (Preferred Reporting Items for Systematic Reviews and Meta-Analyses) guidelines for systematic reviews. After applying the inclusion and exclusion criteria, we identified 41 eligible studies using different states and blood-typing methods to determine blood groups in dogs. The dog blood groups that were identified between 1999 and 2020 in 17 different countries were combined to yield the DEA (Dog Erythrocyte Antigen), Kai, and Dal groups. These studies were conducted in Europe, America, Africa, and Asia but not in all the countries of these continents. The methods used to determine blood types have also changed over the years. This systematic review highlights gaps in the literature and should advance future studies synthesizing data with methodological rigor.

## 1. Introduction

Blood transfusions have become an integral part of veterinary medicine. These procedures are used for the lifesaving treatment of critical diseases. In dogs, as in other species, the presence of glycolipids and glycoproteins on the surfaces of red blood cells allows the classification into blood groups [1,2,3,4,5]. These antigens can cause a reaction via the production of circulating anti-erythrocyte antibodies in a non-compatible host or donor [6]. Over the years, different blood groups have been reported in dogs, and the terminology for canine blood groups has changed over time [7,8,9,10,11]. The acronym DEA is used today for “Dog Erythrocyte Antigen”, followed by the numerical designation of the blood group classified with polyclonal alloantibodies. Five DEA types have been recognized internationally: DEA 1, 3, 4, 5, and 7. However, there are still different opinions regarding the presence of clinically important, naturally occurring alloantibodies against DEA 3, DEA 5, and DEA 7 [12,13,14,15]. In general, the prevalence of DEA 1 in the canine population is about 60% [12]. Within the DEA 1 system, three antigens (1, 2, and 3, or 1.1, 1.2, and 1.3) have been described, but recent studies have described DEA 1 as the dominant autosomal model for classifying dogs as DEA 1-positive or negative [7,15]. Blood antigens other than DEA have also been identified in dogs. In 2007, the Dal blood group was identified in an anemic Dalmatian dog through the use of a gel agglutination assay [13]. The Dal blood group is characterized by anti-*Dal* alloantibodies; Dal is an antigen in red cells associated with anti-Dal alloantibody production [10,13,14,15,16]. Another blood group involves the Kai systems produced by mouse hybridoma techniques [16,17]. Kai was studied in South Korea via the use of monoclonal antibodies, anti-Kai 1, and anti-Kai 2 [16,17,18]. The clinical roles of these blood groups (Dal and Kai) in transfusion treatments still remain to be determined. Pre-transfusion testing is, moreover, used to minimize the risk of immediate or delayed adverse reactions. Blood typing is an important procedure for preventing the induction of alloantibodies against RBCs, reducing the potential for an adverse reaction [9]. Canine blood-typing involves different methods that have been developed, from the Michigan State University test, tube test, and card test to immune-chromatographic strips, cartridges, and flow cytometry assays [19,20]. The principle of all the methods involves the agglutination reaction between red blood cell antigens and monoclonal or polyclonal antisera [20]. The first method described in 1999 was the Michigan State University test (MSU), in which polyclonal antiserum reagents against DEA 1, DEA 3, DEA 4, DEA 5, and DEA 7 were used [21,22]. Other methods for the determination of blood groups were provided for the use of monoclonal sera [22,23,24,25,26]. According to our bibliographic research, the first reported method featuring the use of monoclonal sera is RapidVet-H, which uses the agglutination reaction between the DEA 1.1 antigen and a monoclonal antibody to classify dogs as either positive or negative for DEA 1.1. [22]. In 2005, the typing card test and the gel column method were reported—the first as an agglutination-based reaction featuring the use of monoclonal antibodies [20], and the second based on agglutination in micro-columns using DEA 1.1 monoclonal antibodies within a gel matrix [20,23]. The tube test was used in 2006 with four monoclonal antibodies [24], and in 2011, flow cytometry was reported as a new method for hematology—not only to identify cell phenotypes but also to detect antibodies via the monoclonal murine anti-DEA 1 antibody [12,23,25]. Studies demonstrated that the flow cytometry technique has high specificity and sensitivity compared to other methods [12,19,23,25,26]. Other methods subsequently developed for blood-group determination include the automated canine cartridge dog erythrocyte antigen (DEA) 1.1 blood-typing method (QuickVet/RapidVet) [27] and the immunochromatographic test, which is based on the migration of red blood cells on a membrane and the reaction with monoclonal antibodies specific to each antigen, where a positive result is characterized by the presence of a red line in front of DEA 1 [12,15,19,28]. Recently, new card agglutination tests for DEA 4 and DEA 5 determination were introduced as a quick method [16]. Several studies have involved healthy or hospitalized dogs, as well as different breeds and ages, showing the incidence of blood antigens in dogs by geographic region.

Systematic reviews differ from traditional reviews in several ways. Traditional reviews tend to be mainly descriptive, do not involve a systematic search of the literature, and thereby often focus on a subset of studies in an area chosen based on availability or the author’s selection. Systematic reviews, as their name implies, typically involve a detailed and comprehensive plan and search strategy derived a priori, with the goal of reducing bias by identifying, appraising, and synthesizing all relevant studies on a particular topic [29,30,31,32].

The aim of this review was to summarize data from previously published reports about blood group determination in dogs from different countries following PRISMA guidelines. In this manuscript, a systematic literature review and data assembly were performed to identify relevant studies published between 1999 and 2020 in databases on the geographical distribution of dog blood groups around the world and the varieties of blood-typing assays used. The search included published papers concerning blood groups in various countries, blood groups in different breeds, and blood-typing methods [31]. The purpose was to obtain the most comprehensive information available to determine the prevalence of canine blood groups around the world and the blood-typing methods used over time.

## 2. Materials and Methods

### 2.1. Search Strategy

This retrospective and documental study was conducted with a comprehensive literature review performed using the PubMed and ScienceDirect databases. To refine the studies about blood groups in dogs in different countries, a combination of descriptors, (blood group” OR “DEA” OR “Kai” OR “Dal”) AND (“dog” OR “dogs” OR “canine”), was used. Potential additional references were manually searched for by screening the references of selected articles and the bibliographic sections within transfusion medicine textbooks. For all the selected studies, the following data were extracted: the year of publication, country of study, assay for blood typing, breeds of the dogs, and geographical origins of the dogs.

### 2.2. Study Selection

**Figure 1:**
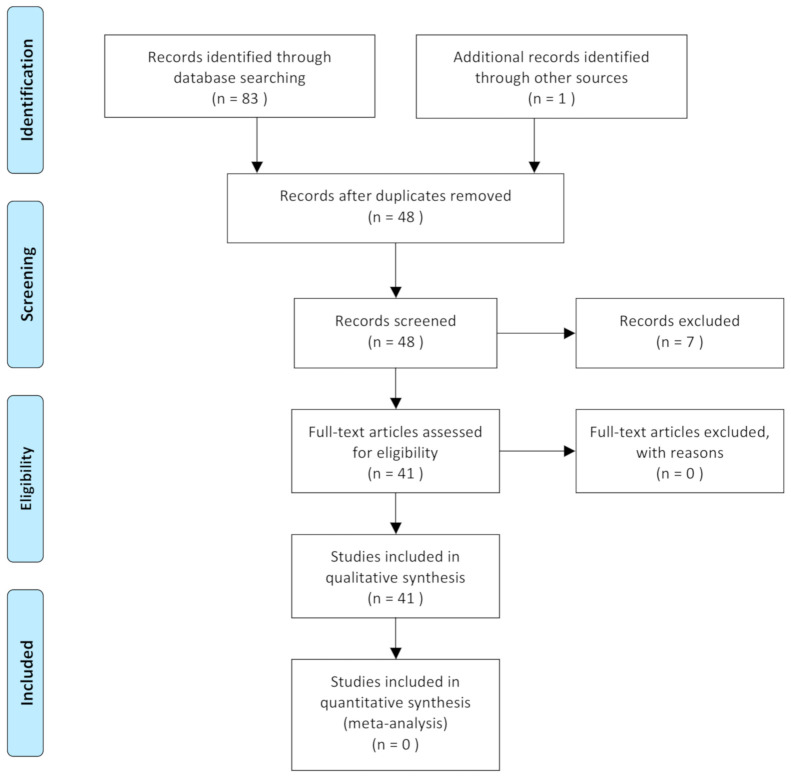
Study selection flowchart for this systematic review (a color figure can be viewed at http://prisma-statement.org/PRISMAStatement/FlowDiagram.aspx).

The inclusion criteria included English-language articles that provided descriptions of typing blood groups in dogs, the use of different canine blood-typing methods, the prevalence of dog erythrocyte antigens in a given country, or the prevalence of dog erythrocyte antigens in a breed. The exclusion criteria included studies with insufficient information regarding the country of the study and studies involving other species. Studies that involved both dogs and cats were not excluded, but data were extracted. This literature review followed the PRISMA (Preferred Reporting Items for Systematic reviews and Meta-Analyses) flowchart and is based on PRISMA’s statement (Figure 1).

After the research strategy was applied, the full texts of the identified articles were assessed to produce the final selection of articles included for this systematic review.

### 2.3. Data and Quality Assessment

As previously anticipated, the following data were recorded from each study: the year of publication, the country where the dogs were enrolled, the numbers of dogs included, the prevalence of specific dog erythrocyte antigens, and the blood-typing method used. The selected studies were divided into 3 groups—DEA, Dal, and Kai—representing the blood groups. The selected studies were also divided into 9 groups based on the blood-typing assay: the Serology Laboratory of Michigan State University (MSU) method, the serological agglutination reaction (RapidVet-H, Canine DEA 1.1., dms/Agrolaboproducts ag Neuhausen am Rheinfall, Switzerland), the typing card test for DEA 1, the tube agglutination test, the gel column method, the immunochromatographic test, flow cytometry, automated canine cartridge dog erythrocyte antigen (DEA) 1.1. detection, and the new card agglutination tests for DEA 4 and DEA 5. The referenced studies were found during the search for articles in the digital databases and additional records from other sources.

## 3. Results

### 3.1. Blood Groups

**Figure 2:**
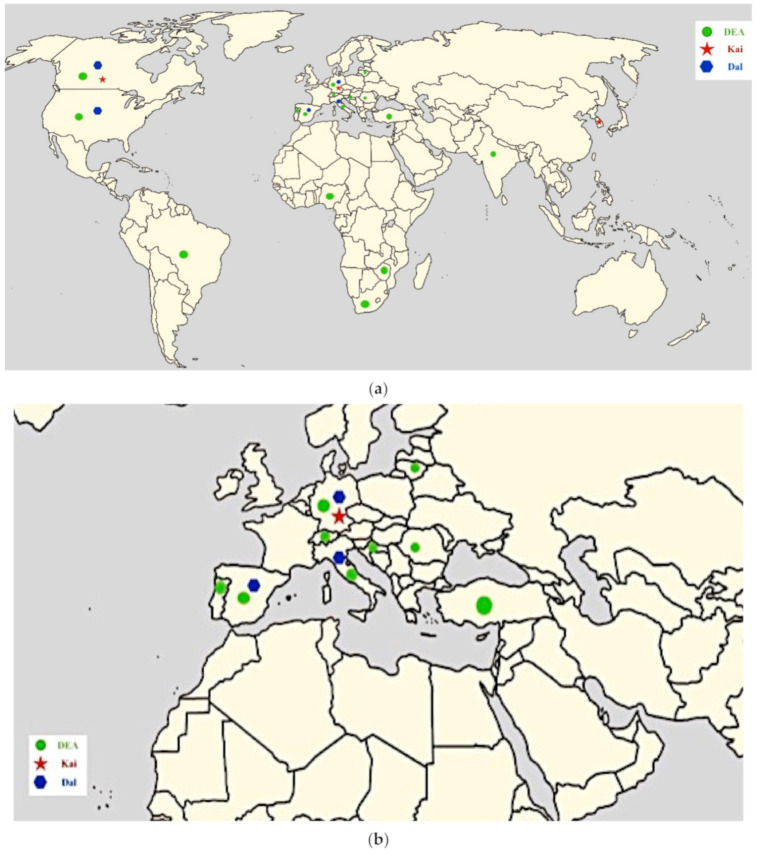
(**a**) A map showing the countries in the world where studies on the determination of dog blood groups (DEA, Kai, and Dal) were carried out; (**b**) A map showing the countries in central Europe where studies on the determination of dog blood groups (DEA, Kai, and Dal) were carried out.

After the application of the inclusion and exclusion criteria, duplicates were removed, and twenty-seven eligible studies on the determination of canine blood groups were identified in different countries. Between 1999 and 2020, the blood groups of mixed-breed dogs were identified in 17 countries: South Africa, Portugal, Turkey, Switzerland, Brazil, Nigeria, India, Spain, North America (Canada, Pennsylvania, New Jersey, Delaware), the USA, South Korea, Zimbabwe, Romania, Croatia, Lithuania, Italy, and Germany (Figure 2a,b).

As shown in the map, between 1999 and 2020, canine blood groups were not determined in all countries around the world. On the American continent, relevant studies were carried out in three countries (the USA, Canada, and Brazil). On the European continent, studies on blood groups were carried out in eight countries (Portugal, Spain, Italy, Switzerland, Croatia, Germany, Romania, and Lithuania). On the African continent, studies were carried out in three countries (Nigeria, South Africa, and Zimbabwe). Finally, on the Asian continent, studies on dog blood group determination were carried out in Turkey, Korea, and India. DEA group results were found to be the most common among all the countries; meanwhile, the Kai group was used in only three countries, and the Dal group, in five countries.

### 3.2. Blood-Typing Methods

After the application of the inclusion and exclusion criteria, duplicates were removed in the selected articles, and 35 eligible studies were identified for different canine blood-typing methods. Some of these articles are the same articles selected to describe the blood groups. Between 1999 and 2020, nine (9) blood-typing methods were described (Table 1).

From 1999 to 2020, the methods used for blood typing in dogs were the same for some blood groups, while new methods were introduced for other specific groups. The Michigan State University test (MSU) was reported in three studies in the USA and Brazil between 1999 and 2011 [20,21,33]. The serological agglutination reaction (RapidVet-H) was reported in nine studies carried out between 2002 and 2019 in Croatia, South Korea, Italy, Romania, Spain, South Africa, Canada, Italy, and the USA [13,17,22,34,35,36,37,38,39]. The typing card for determining DEA 1 was reported in one study in 2005 carried out in the USA [20]. The gel column method was used in 15 studies carried out in the USA, Germany, Turkey, Portugal, North America, Italy, Spain, and Switzerland between 2005 and 2020 [10,13,14,15,16,18,20,27,28,37,40,41,42,43]. The tube agglutination test was reported between 2006 and 2020 in nine studies in Turkey, the USA, Nigeria, Romania, Spain, Canada, and Brazil [10,23,24,36,38,44,45,46,47]. Flow cytometry was used in five studies performed in the USA and Brazil between 2014 and 2020 [12,19,23,25,26]. The automated canine cartridge dog erythrocyte antigen (DEA) 1.1 test (QuickVet/RapidVet) was reported in only one study in Germany in 2012 [27]. The immune-chromatographic test was used between 2013 and 2020 in 15 studies carried out in Italy, India, the USA, Zimbabwe, Germany, North America, Spain, Lithuania, and Brazil [12,15,16,18,19,26,28,46,47,48,49,50,51]. Finally, new card agglutination tests for DEA 4 and DEA 5 were reported in only one study in 2020 in Germany [16].

### 3.3. Prevalence of Blood Groups in Different Dog Breeds and Countries

The prevalence of blood groups in different breeds of dogs from 34 datasets is reported in Table 2.

DEA 1 positivity was determined in some studies in mixed-breed dogs, Ibizan hounds, greyhounds, Nigerian Indigenous dogs, Galgos dogs, Posavaz hounds, and Tornjak hounds with prevalence between 39.89 and 91.3%. DEA 3 was determined in two studies on mixed-breed dogs with prevalence between 10.6 and 23.2%. The DEA 4 blood group was studied mainly in mixed-breed dogs and Ibizan hounds, with prevalence between 98.8 and 100%. DEA 7 was determined in mixed-breed dogs, Ibizan hounds, and Spanish greyhounds, with prevalence between 4.7 and 71.7%. Dal positivity was studied in dalmatians, D. Pinschers, shih tzus, and mixed-breed dogs, with prevalence between 2.2 and 100%. DEA 5, Kai 1, and Kai 2 positivity was determined only in mixed-breed dogs, with prevalence between 1 and 96.6%.

## 4. Discussion

In the last two decades, there have been numerous studies on the determination of blood groups in dogs and the use of different methods for blood group typing. The scientific articles included in this systematic review were published from 1999 to 2020, as described in Table 1 and Table 2, although some studies were also conducted previously (51-2). Transfusion medicine in veterinary medicine has made progress, and it has become clear that the immune-hematology of blood groups plays a fundamental role in such medicine. The determination of blood groups was reported mainly in studies carried out in Europe (Portugal, Spain, Italy, Switzerland, Germany, Croatia, Romania, and Lithuania) and the American continent (North America, the USA, and Brazil). In smaller numbers, studies on blood typing in dogs were carried out in Asia (South Korea, India, and Turkey) and Africa (South Africa, Zimbabwe, and Nigeria). The first method described in the selected articles was the immuno-hematology method at the Serology Laboratory of Michigan State University (MSU) in 1999 [21]. The most recent studies from 2011 and 2020 instead used flow cytometry and immunochromatographic tests [12,15,16,18,19,23,25,26,28,35,39,46,47,48,49,50,51]. However, in some articles, the typing methods used were described differently, even those based on the same method. This made it difficult to refer to a single method.

DEA 1 is the blood group that most often elicits adverse immunological reactions and is responsible for transfusion reactions [52]. For this reason, from past to present, the DEA 1 blood group has been the most commonly studied in all countries, with prevalence rates ranging from 13.1% in greyhounds [43] to 87% in mixed breeds [12]. The studies analyzed in this systematic review showed a discrepancy in the denomination of DEA 1 groups over the years, such as DEA 1. 1 and DEA 1. 2. However, to date, the official classification has provided for the use of DEA 1 as the only blood group [7,15]. Among the pure breeds studied, greyhounds had the lowest prevalence of positivity for DEA 1 (13%) [43], while Ibizan hounds had the highest prevalence (75%) [15]. The DEA 4 blood group was studied in mixed-breed and Ibizan hound dogs. Moreover, the prevalence of DEA 4 blood ranged from 98.8% [35] to 100% [14,15,16] and was thus non-significant during the blood transfusion. The prevalence of positivity for Kai 1 was higher (from 2.94 to 96.6%) than that for Kai 2 (from 1 to 2.9%) [16,18].

## 5. Conclusions

Systematic reviews are the reference standard for synthesizing data because of their methodological rigor. To our knowledge, this is the first review conducted on the prevalence and geographical distribution of dog blood groups. A systematic review is a method used in human medicine to synthesize the current knowledge on targeted issues—for example, to address the status of a disease’s prevalence, etiology, and diagnostic test accuracy and to evaluate preventive or therapeutic interventions. The study of canine blood groups in transfusion began in the 1600s, and from 1937, the presence of six canine blood groups has been further defined [53,54,55]. Today, blood transfusion in dogs is an important practice in veterinary medicine for minimizing the risk of immediate or delayed adverse reactions. Many studies have aimed to identify the prevalence of blood groups in particular geographical areas through the use of different methods. This systematic review summarized the current state of knowledge on blood group determination in countries around the world with reference to the DEA, Kai, and Dal groups and their prevalence, as well as the identification methods used for determination between 1999 and 2020. The studies showed that dog blood typing is possible only via specific laboratory methods, thereby limiting its applicability in clinical practice. For this reason, the existence of rapid commercial kits available from the mid-2000s (Quick Test DEA 1.1 Alvedia^®^, Lyon, France) for the determination of the DEA 1 group [55] makes this group the most widely used in clinical practice. This is different for the DEA3, DEA5, DEA7, Dal, and Kai blood groups noted in scientific studies, which are impractical for blood typing in clinical medicine due to the lack of a quick-to-use test kit. Many studies have compared the accuracy and clinical use of blood-typing methods [27,28,56]. In a study conducted in 2012, the gel-based method was found to be the most accurate (100%) when compared to the card agglutination assay, immunochromatographic cartridge method, and gel method for DEA 1.1 blood typing [56]. The test sensitivity can be considered the most important test characteristic when screening patients in emergency or routine situations. Moreover, new agglutination cards (Card, the RapidVet-H DEA 4 Agglutination Card Test, and the DEA 5 Card RapidVet-H DEA 5 Agglutination Card Test, DMS, Flemington, NJ, USA) have recently become commercially available for use with polyclonal anti-DEA 4 or anti-DEA 5 antisera [16]. Studies on the use of this rapid method for the detection of DEA 4 and DEA 5 blood groups in the clinical practice of transfusion medicine are still rare. In conclusion, this systematic review will be useful for suggesting gaps in the literature and advancing future research.

## Figures and Tables

**Table 1:** Methods used to determine canine blood groups in different countries. All these studies were published between 1999 and 2020.

Table 2005	Country	Reference
MSU Michigan State University test	USA Brazil	Giger et al. (2005) [20] Novais et al. (1999) [21] Esteves et al. (2011) [33]
Serological agglutination reaction, RapidVet-H (Canine DEA 1.1., dms/Agrolaboproducts ag Neuhausen am Rheinfall, Switzerland)	Croatia	Živčić et al. (2013) [34]
	South Korea	Lee et al. (2017) [17]
	Italy	Medina Valentin et al. (2017) [35]
	Romania	Ognean (2014) [36]
	Spain	Spada et al. (2016) [37]
	South Africa	van der Merwe et al. (2002) [22]
	Canada	Villarnovo et al. (2016) [38]
	Italy	Proverbio et al. (2019) [39]
	USA	Blais et al. (2007) [13]
Typing card test for DEA 1	USA	Giger et al. (2005) [20]
Gel column method	USA	Blais et al. (2007) [13]
		Blois et al. (2013) [28]
	Germany	Kohn et al. (2012) [27]
		Ebelt et al. (2020) [16]
	Turkey	Ergul Ekiz et al. (2011) [40]
	Portugal	Ferreira et al. (2011) [41]
	North America	Giger et al. (2005) [20]
		Kessler et al. (2010) [10]
		Blois et al. (2013) [28]
		Euler et al. (2016) [18]
		Goulet et al. (2017) [42]
		Goulet et al. (2018) [14]
	Italy	Proverbio et al. (2020) [15]
	Spain	Spada et al. (2016) [37]
	Switzerland	Riond et al. (2011) [43]
Tube test	Turkey	Arikan et al. (2009) [44]
	USA	Iazbik et al. (2010) [45]
		Kessler et al. (2010) [10]
		Lucidi et al. (2011) [23]
	Nigeria	Nottidge et al. (2006) [24]
	Romania	Ognean (2014) [36]
	Spain	Spada et al. (2018) [46]
	Canada	Villarnovo et al. (2016) [38]
	Brazil	Santos et al. 2020 [47]
Flow cytometry		Lucidi et al. (2011) [23]
	USA	Acierno et al. (2014) [12]
		Polak et al. (2015) [19]
	Brazil	Santos et al. (2018) [25]
		Santos et al. (2020) [26]
Automated canine cartridge dog erythrocyte antigen (DEA) 1.1 blood-typing method (QuickVet/RapidVet)	Germany	Kohn et al. (2012) [27]
Immunochromatographic test	Italy	Carli et al. (2017) [47]
		Medina Valentin et al. (2017) [35]
		Proverbio et al. (2019) [39]
		Proverbio et al. (2020) [15]
	India	Baranidharan et al. (2018) [48]
	USA	Acierno et al. (2014) [12]
		Polak et al. (2015) [19]
	Zimbabwe	Dhliwayo et al. (2016) [49]
	Germany	Ebelt et al. (2020) [16]
	North America	Blois et al. (2013) [28]
		Euler et al. (2016) [18]
	Spain	Mesa-Sanchez et al. (2014) [50]
		Spada et al. (2018) [46]
		Proverbio et al. (2020) [15]
	Lithuania	Paleckaitis et al. (2018) [51]
	Brazil	Santos et al. 2020 [26]
New card agglutination tests for DEA 4 and DEA 5	Germany	Ebelt et al. (2020) [16]

**Table 2:** Prevalence of blood groups (DEA 1, DEA 3, DEA 4, DEA 5, DEA 7, Dal, Kai1, and Kai2) in dogs.

Blood Groups. Positive	Breed	Number of Dogs n (%)	Reference
DEA 1	Mixed Breed	172 (59%)	Lucidi et al. (2011) [23]
	Mixed Breed	66 (87%)	Acierno et al. (2014) [12]
	Mixed Breed	96 (55%)	Kohn et al. (2012) [27]
	Mixed Breed	206 (59.2%)	Ebelt et al. (2020) [16]
	Mixed Breed	178 (65.2%)	Ergul Ekiz et al. (2011) [40]
	Mixed Breed	198 (61.1%)	Arikan et al. (2009) [44]
	Mixed Breed	274 (56.9%)	Ferreira et al. (2011) [41]
	Mixed Breed	43 (46.5–58%)	Kessler et al. (2010) [10]
	Mixed Breed	503 (59.6%)	Euler et al. (2016) [18]
	Mixed Breed	320 (42.8%)	Proverbio et al. (2020) [15]
	Ibizan Hounds	92 (75%)	Spada et al. (2016) [37]
	Mixed Breed	304 (50%)	Riond et al. (2011) [43]
	Greyhound	206 (13.1%)	Iazbik et al. (2010) [45]
	Mixed Breed	66 (60.6%)	Iazbik et al. (2010) [45]
	Nigerian Indigenous	178 (39.89%)	Nottidge et al. (2006) [24]
	Mixed Breed	1037 (62%)	Carli et al. (2017) [47]
	Mixed Breed	7414 (61.2%)	Medina Valentin et al. (2017) [35]
	Mixed Breed	125 (61.6%)	Baranidharan et al. (2018) [48]
	Mixed-Breed	100 (78%)	Dhliwayo et al. (2016) [49]
	Galgos	88 (55.7%)	Mesa-Sanchez et al. (2014) [50]
	Mixed Breed	118 (51.7%)	Mesa-Sanchez et al. (2014) [50]
	Mixed Breed	233 (47%)	van der Merwe et al. (2002) [22]
	Mixed Breed	150 (91.3%)	Novais et al. (1999) [21]
	Posavaz Hound	30 (60%)	Živčić et al. (2013) [34]
	Tornjak Hounds	30 (53.3%)	Živčić et al. (2013) [34]
	Mixed Breed	100 (83%)	Esteves et al. (2011) [33]
	Mixed Breed	69 (56%)	Santos et al. 2020 [26]
DEA 3	Mixed Breed	198 (23.2%)	Arikan et al. (2009) [44]
	Mixed Breed	75 (10.6–13.3%)	Kessler et al. (2010) [10]
	Mixed Breed	100(7%)	Esteves et al. (2011) [33]
DEA 4	Mixed Breed	206 (100%)	Ebelt et al. (2020) [16]
	Mixed Breed	198 (100%)	Arikan et al. (2009) [44]
	Mixed Breed	75 (100%)	Kessler et al. (2010) [10]
	Mixed Breed	320 (100%)	Proverbio et al. (2020) [15]
	Ibizan Hounds	92 (98.8%)	Spada et al. (2016) [37]
	Mixed Breed	100 (100%)	Esteves et al. (2011) [33]
DEA 5	Mixed Breed	206 (9–11%)	Ebelt et al. (2020) [16]
	Mixed Breed	198 (55.5%)	Arikan et al. (2009) [44]
	Mixed Breed	100 (9%)	Esteves et al. (2011) [33]
DEA 7	Mixed Breed	198 (71.7%)	Arikan et al. (2009) [44]
	Mixed Breed	75 (12–22.6%)	Kessler et al. (2010) [10]
	Mixed Breed	320 (13.4%)	Proverbio et al. (2020) [15]
	Ibizan Hounds	92 (25%)	Spada et al. (2016) [37]
	Spanish Greyhounds	42 (4.7%)	Spada et al. (2018) [46]
	Mixed Breed	100 (16%)	Esteves et al. (2011) [33]
Dal	Mixed Breed	206 (89.3%)	Ebelt et al. (2020) [16]
	Mixed Breed	63 (100%)	Kessler et al. (2010) [10]
	Dalmatians	128 (85.5–100%)	Goulet et al. (2017) [42]
	D. Pinschers	432 (43.3–78.6%)	Goulet et al. (2017) [42]
	Shih Tzus	21 (21.4–100%)	Goulet et al. (2017) [42]
	Mixed Breed	549 (98.6–100%)	Goulet et al. (2017) [42]
	Mixed Breed	320 (2.2%)	Proverbio et al. (2020) [15]
Kai 1	Mixed Breed	206 (96.6%)	Ebelt et al. (2020) [16]
	Mixed Breed	503 (2.94%)	Euler et al. (2016) [18]
Kai 2	Mixed Breed	206 (2.9%)	Ebelt et al. (2020) [16]
	Mixed Breed	503 (1%)	Euler et al. (2016) [18]

## Data Availability

Not applicable.
